# Parathyroid hormone is a plausible mediator for the metabolic syndrome in the morbidly obese: a cross-sectional study

**DOI:** 10.1186/1475-2840-10-17

**Published:** 2011-02-10

**Authors:** Jo Røislien, Ben Van Calster, Jøran Hjelmesæth

**Affiliations:** 1 Department of Biostatistics, Institute of Basic Medical Sciences, University of Oslo, Norway; 2 Department of Electrical Engineering (ESAT-SISTA), Katholieke Universiteit Leuven, Belgium; 3 Morbid Obesity Center, Vestfold Hospital Trust, Tønsberg, Norway

## Abstract

**Background:**

The biological mechanisms in the association between the metabolic syndrome (MS) and various biomarkers, such as 25-hydroxyvitamin D (vit D) and magnesium, are not fully understood. Several of the proposed predictors of MS are also possible predictors of parathyroid hormone (PTH). We aimed to explore whether PTH is a possible mediator between MS and various possible explanatory variables in morbidly obese patients.

**Methods:**

Fasting serum levels of PTH, vit D and magnesium were assessed in a cross-sectional study of 1,017 consecutive morbidly obese patients (68% women). Dependencies between MS and a total of seven possible explanatory variables as suggested in the literature, including PTH, vit D and magnesium, were specified in a path diagram, including both direct and indirect effects. Possible gender differences were also included. Effects were estimated using Bayesian path analysis, a multivariable regression technique, and expressed using standardized regression coefficients.

**Results:**

Sixty-eight percent of the patients had MS. In addition to type 2 diabetes and age, both PTH and serum phosphate had significant direct effects on MS; 0.36 (95% Credibility Interval (CrI) [0.15, 0.57]) and 0.28 (95% CrI [0.10,0.47]), respectively. However, due to significant gender differences, an increase in either PTH or phosphate corresponded to an increased OR for MS in women only. All proposed predictors of MS had significant direct effects on PTH, with vit D and phosphate the strongest; -0.27 (95% CrI [-0.33,-0.21]) and -0.26 (95% CrI [-0.32,-0.20]), respectively. Though neither vit D nor magnesium had significant direct effects on MS, for women they both affected MS indirectly, due to the strong direct effect of PTH on MS. For phosphate, the indirect effect on MS, mediated through serum calcium and PTH, had opposite sign than the direct effect, resulting in the total effect on MS being somewhat attenuated compared to the direct effect only.

**Conclusion:**

Our results indicate that for women PTH is a plausible mediator in the association between MS and a range of explanatory variables, including vit D, magnesium and phosphate.

## Background

The metabolic syndrome (MS) is a clustering of risk factors including abdominal obesity, insulin resistance and elevated blood pressure [1], and it has an increased incidence among the obese part of the population; a proportion which is steadily growing [2,3]. Having the MS is associated with increased risk of type 2 diabetes, cardiovascular disease [4] and chronic kidney disease [5]. A better understanding the possible biological mechanisms related to the occurrence of MS is therefore of major interest.

Age and diabetes are well-known to be associated with MS [6]. A number of studies indicate that MS is also related to various biomarkers such as serum 25-hydroxyvitamin D (vit D), magnesium and calcium [7-11]. Elevated levels of parathyroid hormone (PTH) have been reported together with MS [12,13]. In studies focusing on other variables, it has been debated whether these variables should be adjusted for PTH in the analyses [14-16]. Indeed, the reported effects of PTH are contradictory. A recent publication on morbidly obese subjects found a relationship between MS and PTH [17], which is in contrast with a previous study [18].

Establishing the role of PTH in relation to MS is complicated by the fact that several of the proposed predictors of MS are also known to be associated with PTH. The synthesis and secretion of PTH is tightly regulated by serum levels of calcium and phosphate, but also serum concentrations of vit D and magnesium influence PTH levels [19,20], and a statistically significant negative correlation between vit D and PTH has been reported [17]. Studies further indicate that gender differences should be taken into account; increasing PTH levels have been reported to be associated with MS in older men, but not in women and younger men [12,13].

The mechanisms explaining the association between MS and various biomarkers are not well understood, and focusing solely on direct effects might overlook clinically interesting indirect effects. The above references indicate that PTH might be a mediator for MS, highlighting the need for analytical approaches that include both direct and indirect effects.

The aim of this study was to explore whether PTH might have a mediating role in the association between MS and a series of possible explanatory variables in morbidly obese patients. Dependencies between MS and a series of variables suggested in the literature were depicted in a path diagram. Effect sizes were estimated by path analysis, a regression technique where several multiple regression models are combined, allowing variables to be both an outcome and an explanatory variable in the same model [21].

## Methods

### Study population

Individuals having BMI ≥40 kg/m², or a BMI ≥35 kg/m² with at least one obesity-related comorbidity, are defined as being morbidly obese. The data set used for the present analysis was a cross-sectional study of 1,017 morbidly obese Caucasian individuals, collected prospectively between November 28^th^, 2005 and September 16^th^, 2008, at the Morbid Obesity Centre in the county of Vestfold, Norway. The study was approved by the Regional Committee for Medical Research Ethics (S-05175) and was performed in accordance with the Declaration of Helsinki [22].

MS was diagnosed in patients with at least three of the following characteristics [1]; elevated waist circumference (≥102 cm in men and ≥88 cm in women), elevated fasting triglycerides (≥1.7 mmol/l), elevated blood pressure, elevated fasting glucose (≥5.6 mmol/l) or diabetes, or reduced HDL-cholesterol (<1.0 mmol/l in men and <1.3 mmol/l in women). Type 2 diabetes was diagnosed in patients who had a prior history of type 2 diabetes or a fasting serum glucose level ≥7.0 mmol/l [23].

The data has been published previously, and further details on definitions, physical examination and laboratory analyses can be found elsewhere [17].

### Path model

**Figure 1:**
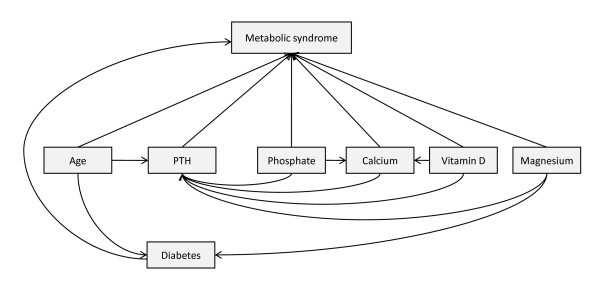
**Path diagram showing hypothesized relationships between variables, decomposed into direct and indirect effects**. Path diagram showing hypothesized relationships between variables as suggested in the literature, decomposed into direct and indirect effects. The indirect pathways between the included explanatory variables and MS were hypothesized to be mediated by PTH. Arrows represent dependencies between variables. Absence of an arrow between two variables indicates that the variables are considered to be statistically independent in the model.

Based on previous research the following seven explanatory variables were included in a statistical model for the occurrence of MS; age, type 2 diabetes (T2DM), PTH, vit D, calcium, phosphate and magnesium. The relations between the variables, as suggested in the literature, are summarized in a path model (Figure 1). The effects of the explanatory variables are decomposed into a direct effect on MS and various possible indirect effects, mediated by among others PTH. The proposed path model is based on the following.

It is well known that age is associated with an increased odds of MS and T2DM, while T2DM is associated with increased risk of MS [6,17]. Low serum magnesium has been shown to be a predictor of both MS and T2DM [10,17,24,25] while low phosphate and increased levels of albumin-corrected calcium is related to increased incidence of MS [25-27]. Vit D is also associated with MS [7,8,11].

PTH has been shown to be associated with increased odds for MS, even after adjustment for vit D [17]. The synthesis and secretion of PTH is regulated by serum levels of calcium and phosphate [20], while obesity, older age and reduced levels of calcium and vit D are all associated with higher levels of PTH [20,28,29]. Studies have shown that serum concentrations of vit D and magnesium influence PTH levels [19,20], while low serum magnesium has been shown to inhibit PTH secretion [19]. Finally, both vit D and phosphate are associated with calcium [20].

Arrows in a path diagram represent suggested dependencies between variables, and absence of an arrow between two variables indicates that these variables are considered statistically independent in the model. All hypothesized direct and indirect relations among measured variables can be read off the path diagram.

### Statistical analysis

Descriptive statistics are presented as mean and standard deviation (SD) or frequency and percentage (%). Chi square and independent samples T-tests were used for univariate comparisons.

The parameters of our proposed path model (Figure 1) were estimated using path analysis. Analytical methods in clinical research often rely on multiple regression models with one main outcome variable, and a set of explanatory variables treated on equal terms. Path analysis, in contrast, is a multivariable method based on a model with several linked regression equations [21]. Within this system of equations, some of the variables can be considered both as outcome variables and as explanatory variables at the same time. Path analysis is a form of structural equation modeling, and requires that all hypothesized dependencies between the variables are specified in a model and depicted in a path diagram prior to the analysis.

The path diagram formed the basis for the path analysis, in which we obtain direct and indirect effect estimates simultaneously by combining four regression equations, each with one of the following variables as the outcome: MS, PTH, calcium and T2DM. In order to assess the validity of the underlying assumption of linearity in the separate linear and logistic regressions, generalized additive models (GAM) [30] were fitted prior to the path analysis.

We also wanted to test for gender differences. As a preliminary analysis, we therefore fitted the path model separately for men and women. This analysis suggested possible gender differences for several paths, and parameters for such possible gender effects were included in the full model and estimated.

All continuous variables were standardized prior to the analysis. Standardization allows one to compare the relative importance of various effects in a model, whereas using original variables allows for comparison across studies [31]. We mainly report results as standardized regression coefficients, but include the unstandardized coefficients for comparison. For binary outcomes the corresponding regression coefficients equal the logarithm of the odds ratio (OR).

For pathways consisting of only continuous variables, e.g. calcium→PTH→MS, indirect effects can be found by multiplication of the regression coefficients along this pathway, while total effects can be found by summing effects along each separate pathway connecting two variables, e.g. calcium→MS plus calcium→PTH→MS. When binary outcomes are involved somewhere along a pathway, the situation is less straightforward; regression coefficients have different interpretations in linear and logistic regression, and merely adding and multiplying regression coefficients is not meaningful.

Statistical analyses were performed in R 2.11 [32]. The path analyses were done using Bayesian estimation procedures [33] by running WinBUGS [34] from within R. Bayesian estimation gives estimates of regression coefficients and corresponding credibility intervals (CrIs), which are comparable to frequentistic confidence intervals. Considerations of statistical significance were based on the coverage of the credibility intervals. Comparison of path models was done by the deviance information criterion (DIC); lower numbers of DIC are preferable [35]. Further details of the Bayesian model specification and model fitting can be found in Appendix A.

## Results

On the variables in our path model we had complete observations for 971 (95.5%) patients, which were included in the statistical analyses. Clinical and biochemical characteristics are summarized in Table 1. There were 655 (67.5%) women, and all men and women had a waist circumference >102 cm and >88 cm, respectively. In total 662 (68.1%) had MS (65.4% and 73.3% for women and men, respectively, p = 0.013). Of the 246 patients who had T2DM 241 (98%) also had MS.

**Table 1:** Characteristics of patients according to gender

		Gender		
			
Variables Variables	Total	Female	Male	P
Number of patients	971	655	316	
Age (years)	42 (12)	41 (12)	44 (12)	0.001
Waist (cm)	133 (14)	129 (13)	141 (14)	<0.001
MS	661 (68%)	429 (65%)	232 (73%)	0.013
Type 2 diabetes	247 (25%)	147 (22)	100 (32%)	0.002
PTH (pmol/l)	5.8 (2.3)	5.8 (2.3)	5.9 (2.4)	0.597
25(OH)D (nmol/l)	52 (22)	54 (22)	50 (21)	0.009
Magnesium (mmol/l)	0.84 (0.07)	0.84 (0.07)	0.85 (0.07)	0.522
Calcium (mmol/l)	2.35 (0.07)	2.36 (0.07)	2.35 (0.07)	0.024
Phosphate (mmol/l)	1.09 (0.17)	1.10 (0.16)	1.06 (0.17)	<0.001

Data are means (SD) for continuous variables and n (%) for categorical variables.

### Path model estimation

We first performed a path analysis of the model in Figure 1 separately for men and women as a preliminary analysis to explore possible gender differences. Besides an apparent gender difference in the direct effects of age on both PTH and MS, and in the direct effect of PTH on MS, also phosphate showed a possibly significant gender difference in the direct path to MS (data not shown). Parameters for these possible gender differences were included along their respective paths in the overall path model, resulting in a model with reduced DIC compared to a model without such gender differences (from 7182 to 7172). Removing statistically non-significant direct paths from this larger model reduced the DIC further (from 7172 to 7142). Regression results from this final, optimal model, expressed as standardized regression coefficients and CrIs, are shown in Table 2. Unstandardized results are included for reference.

**Table 2:** Bayesian path analysis with gender effects. For a select variables the direct effect is for women only, accompanied by an estimation of significant gender difference and corresponding effect for males. The unstandardized effect estimates are odds ratios (OR) for binary outcomes

VARIABLES		ESTIMATES USING STANDARDIZED VARIABLES			UNSTANDARDIZED EFFECT ESTIMATES	
**Outcome** **variable**	**Explanatory** **Variable**	**Direct effect** **(95% CrI)**	**Gender diff.** **(95% CrI)**	**Corresp. effect for males** **(95% CrI)**	**Direct effect** **(95% CrI)**	**Effect for males** **(95% CrI)**

**MS**^bin^	Diabetes	3.47 (2.65,4.42)			32.0 (14.2,83.4)	
	Age	0.31 (0.13,0.50)	-0.29 (-0.64,0.04)	0.02 (-0.25,0.30)	1.03 (1.01,1.04)	1.00 (0.98,1.03)
	PTH	0.36 (0.15,0.57)	-0.43 (-0.77,-0.11)	-0.07 (-0.32,0.19)	1.17 (1.07,1.28)	0.97 (0.87,1.09)
	Phosphate	0.28 (0.10,0.47)	-0.42 (-0.76,-0.05)	-0.14 (-0.41,0.12)	5.32 (1.79,17.39)	0.42 (0.08,2.10)

**PTH**^c^	Age	0.20 (0.13,0.27)	-0.18 (-0.29,-0.06)	0.03 (-0.07,0.12)	0.04 (0.03,0.05)	0.005 (-0.013,0.023)
	Magnesium	0.12 (0.06,0.18)			3.76 (2.00,5.59)	
	Vitamin D	-0.27 (-0.33,-0.21)			-0.028 (-0.034,-0.022)	
	Phosphate	-0.26 (-0.32,-0.20)			-3.57 (-4.38,-2.81)	
	Calcium*	-0.09 (-0.15,-0.04)			-3.17 (-5.13,-1.19)	

Calcium*^c^	Phosphate	0.27 (0.21,0.33)			1.31 (1.23,1.39)	

**Diabetes**^bin^	Magnesium	-0.76 (-0.93,-0.59)			0.000 (0.000,0.0002)	
	Age	0.88 (0.71,1.07)			1.08 (1.06,1.09)	

^bin ^binary outcome (logistic regression)

^c ^continuous outcome (linear regression)

* Albumin corrected

### MS

Besides diabetes and age, only PTH and phosphate had significant direct effects on MS. The effect of T2DM was by far the strongest, while the estimated direct effect of PTH was 0.36 (95% CrI [0.15,0.57]). This value implies that a 1 SD increase in PTH (2.3 pmol/l) results in an increase of 0.36 in the log odds of MS. Due to a significant gender difference, this direct effect of PTH on MS was significant for women only.

Direct effects of age and phosphate were 0.31 (95% CrI [0.13,0.50]) and 0.28 (95% CrI [0.10,0.47]), respectively, implying that an increase in 1 SD in age or phosphate (12 years and 0.17 mmol/l, respectively) results in an increase in the log OR of MS by approximately one third. Due to significant gender differences, these direct effects on MS were also only significant for women.

Even though the 95% CrI for the gender difference for age on MS included zero, removing this gender effect from the model actually resulted in a model with an increased DIC, arguing for keeping it in the model.

### PTH

All proposed explanatory variables of MS had significant direct effects on PTH. As a consequence, they all have significant indirect effects on MS for women. The strongest direct effects on PTH were vit D and phosphate; -0.27 (95% CrI [-0.33,-0.21]) and -0.26 (95% CrI [-0.32,-0.20]), respectively. This value implies that an increase of 1 SD in vit D or phosphate (22.0 nmol/l and 0.17 mmol/l, respectively) corresponds to a mean decrease of approximately one fourth SD in PTH (-0.58 pmol/l). However, phosphate also has an indirect effect on PTH, mediated through calcium. The direct effect of phosphate on calcium is 0.27 (95% CrI [0.21, 0.33]), while the direct effect of calcium on PTH is -0.09 (95% CrI [-0.15,-0.04]). The total effect of phosphate on PTH is thus -0.26+(-0.09)*0.27 = -0.28; about a 10% increase compared to the direct effect alone. While the effect of phosphate on PTH, and in result MS, is partly mediated through calcium, the effect of magnesium on MS is mediated through both PTH and T2DM. Due to T2DM being a binary outcome along one of the pathways, this total effect cannot be quantified.

The direct effect of age on PTH was 0.20 (95% CrI [0.13,0.27]). For the direct effect of age on PTH there was also a significant gender effect, resulting in this direct effect of age being significant for women only. For women, the total effect of age on MS is thus split into a direct path and two indirect paths; one via T2DM and one via PTH.

### MS, PTH and gender differences

**Figure 2:**
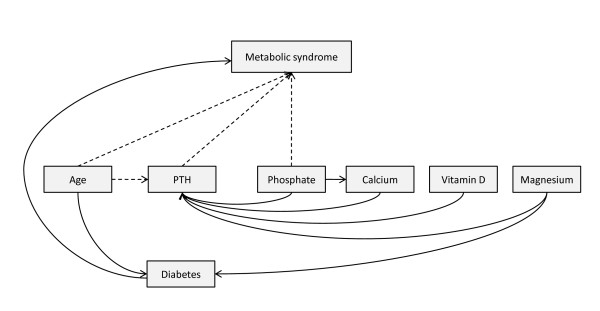
**Path diagram showing the observed direct effects between variables using path analysis**. Path diagram showing statistically significant direct paths in a hypothesized path diagram (Figure 1). Dotted lines imply significant effects for women only.

A summary of the statistically significant paths for men and women is presented in Figure 2, while Table 3 summarizes the direct and indirect effects the various proposed explanatory variables has on MS.

**Table 3:** Type of effect of various explanatory variables on MS

	Type of effect on MS	
Variable	Women	Men
PTH	Direct	-
T2DM	Direct	Direct
Age	Direct and indirect through PTH and T2DM	Indirect through T2DM
Phosphate	Direct and indirect through PTH and calcium	-
Calcium	Indirect through PTH	-
Vitamin D	Indirect through PTH	-
Magnesium	Indirect through PTH and T2DM	Indirect through T2DM

## Discussion

Previous reports have addressed the effect of PTH on MS, and discussed whether the variable should be adjusted for when estimating the effect of other possible predictors of MS [14-17]. Our path analysis on morbidly obese subjects suggests a possible mediating role of PTH in the association between MS and various demographic variables and biomarkers. Significant gender differences indicate, however, that this mediating effect is only significant for women.

### Path model specification

The use of path diagrams and analysis of structural models is a potentially valuable tool for increasing biological understanding [36], and is expanding in the fields of epidemiology and medicine, including studies of MS [37-39].

The crucial task in path analysis is to formulate a plausible path diagram based on existing evidence and current biological concepts. Path analysis is a multivariable regression technique and the hypothesized directions of arrows in the path diagram will thus affect results: Changing the direction of an arrow changes a variable's status from explanatory to outcome, or vice versa.

We recognize that while more complex than traditional multiple regression models, our path model is still only one of many plausible models that can be hypothesized in order to explain the underlying biological mechanisms. There are several possible variables not included in our model that might influence estimates and considerations of direct and indirect effects, for example creatinine [40]. Both alternative path models and expanded versions of the present model, including more and/or other variables and plausible paths, are valuable topics for future research.

### PTH, MS and gender differences

The hypothesized mediating role of PTH in relation to MS was partly confirmed in our data. Of the included explanatory variables, several did not have significant direct paths to MS, while all but one had significant direct paths to PTH, implying significant indirect effects on MS. There was, however, a significant gender difference in the direct path from PTH to MS, implying that this direct effect was only significant for women. The proposed mediating role of PTH is as such a possible property for women only.

The gender specific association between PTH and MS might be explained by some of the properties of the patient population. Men have a higher prevalence of diabetes than women (32% vs 22%) and MS (73% vs 65%) (Table 1). The diabetes prevalence difference might be of particular interest since diabetic patients with MS have lower PTH levels than MS-subjects without diabetes [17]. In addition, the pathophysiology of MS may differ between genders [41]. Male patients also had significantly lower levels of phosphate and calcium.

The increased effect of age on PTH was also only significant for women. For men there was no direct effect on MS of neither PTH nor age. In other studies, increasing PTH levels seem to be associated with MS in older men, but not in women and younger men [12,13,42]. This is contrary to our findings. However, previous studies have not found significant relationships between PTH, vit D and MS in morbidly obese subjects [18].

### PTH as a mediator?

Weight reduction and higher intakes of calcium and vitamin D have been found to be associated with decreases in PTH levels [28,29]. So even though the direct effect of calcium and vitamin D on MS are not significant, both may still have clinical value, given their indirect effects, mediated through PTH. It has been claimed that PTH is associated with MS, while vit D is not [17]. According to our analysis, this only holds true if considering direct effects only. Lowering PTH, for example through medication or through dietary modulation or weight reduction, is potentially a way of reducing the risk of MS; increasing intake of vit D and calcium might indirectly achieve similar results.

Exactly why lowered PTH values are associated with reduced odds of MS is unclear. A recently published link between PTH and MS in older men was explained by insulin resistance, high blood pressure, hyperglycemia and low HDL-cholesterol [12,13]. The hypothesis that PTH may be involved in the pathogenesis of hypertension is also supported by a prospective population based cohort study [43]. The positive correlation between serum PTH and blood pressure has been confirmed [17], but not between PTH and insulin resistance [7], blood glucose or blood lipids [12,13].

Whether lowering of serum PTH does indeed translate into beneficial effects on MS remains unclear, and cannot be assessed in a cross-sectional study; whether PTH is a mediator in the biological processes cannot be confirmed by a study like ours. We want to emphasize the need for experimental studies to assess the molecular mechanisms, as well as the importance of interpreting results from observational studies with caution concerning causality. However, with PTH regulative medications like cinacalcet (Sensipar/Mimpara, Amgen Inc., Thousand Oaks, CA) on the market, a randomized controlled trial can be carried out.

### Phosphate

Lower levels of phosphate have been shown to be associated with the components of MS [44], and are usually related to increased risk of MS [25-27]. This does not appear to be the case in our data of morbidly obese patients. For women increasing levels of phosphate was associated with increased risk of MS. Due to a significant gender difference, there was no direct effect of phosphate on MS for men, nor an indirect effect mediated through PTH. An explanation for this gender difference could be that men had significantly lower phosphate levels than women (Table 1).

### Magnesium, T2DM and MS

Low serum magnesium has been shown to be associated with T2DM and MS [10,17,24,25], but when adjusting for T2DM in multiple regression analyses, the association between magnesium and MS is no longer statistically significant [17]. However, this does not mean that serum magnesium is unrelated to MS. It merely implies that the effects are possibly indirect rather than direct. Our path analysis shows that there is an indirect effect of magnesium on MS, mediated through T2DM; lowering magnesium levels will increase the odds of T2DM which again increases the odds of MS.

### Bayesian analysis

Bayesian methods has only been used to a limited extent in clinical research, but the WinBUGS software has made Bayesian methods more easily available [45]. While traditional frequentistic analyses are based on normality assumptions and central limit theory, Bayesian analyses are based on assumptions of prior distributions and on simulation techniques. In Bayesian models non-normality and non-linearity is easily dealt with. They are also flexible with respect to several types of variables [45].

### Strengths and limitations

The current study is based on a relatively large, homogenous sample of morbidly obese individuals, which is further strengthened by the prospective collection and registration of data. In our model all variables are effectively adjusted for one another, and all parameters estimated simultaneously, reducing the effect of multiple testing.

The study had some limitations. First, the cross-sectional design makes it impossible to establish cause-effect relationships. Though potentially graphically convincing and appealing, a path model cannot itself assess causality. The directions of arrows in the path diagram are a crucial point. For this, micro-level biochemical experiments are needed. Second, the results may not be valid in non-white populations or among non-obese subjects. Finally, we cannot exclude the possibility that referral of patients to a tertiary care center might have introduced a selection bias.

## Conclusions

The results of our study, combining current biological concepts and empirical data, suggest that PTH is a plausible mediator in the association between MS and various demographic variables and biomarkers, including vit D, phosphate and magnesium, in morbidly obese individuals. Due to significant gender differences, in our data this mediating role was only significant for women. Mechanisms like metabolic pathways are complex, and graphical models like the current can be useful tools.

## Competing interests

The authors declare that they have no competing interests.

## Authors' contributions

JR contributed to the conception and design, the statistical analyses, interpretation of data, drafted the manuscript and revised it critically for important intellectual content. BvC contributed to the concept and design, statistical analyses, was involved in drafting the manuscript and revised it critically for important intellectual content. JH contributed to the acquisition of data, statistical analysis and interpretation of data, drafted the manuscript and revised it critically for important intellectual content. All authors read and approved the final manuscript.

## Appendix A

All path estimates presented in this paper were calculated using Markov Chain Monte Carlo simulation (MCMC) in WinBugs [34], run through the R wrapper R2WinBUGS[46] in the freeware R 2.11 [32]. Stochastic simulations in WinBUGS can be unstable, particularly for complex hierarchical models, and diffuse prior distributions add to the problem [47]. We used vague prior probability distributions for all parameters [33], but the common advise of standardizing variables resolved instability issues in our case. We used 3 parallel MCMC chains in the calculations, each based on 10,000 iterations from which the first 5,000 were discarded as a "burn-in". This ensured an estimated potential scale reduction (EPSR) of approximately 1 for all parameters, implying convergence [48].
